# Supplementary figures and images for: Identification of a GrgA-Euo-HrcA Transcriptional Regulatory Network in Chlamydia
Source: mSystems. 2021 Aug 3;6(4):e00738-21. doi: 10.1128/mSystems.00738-21 (PMC8409740; doi:10.1128/mSystems.00738-21)

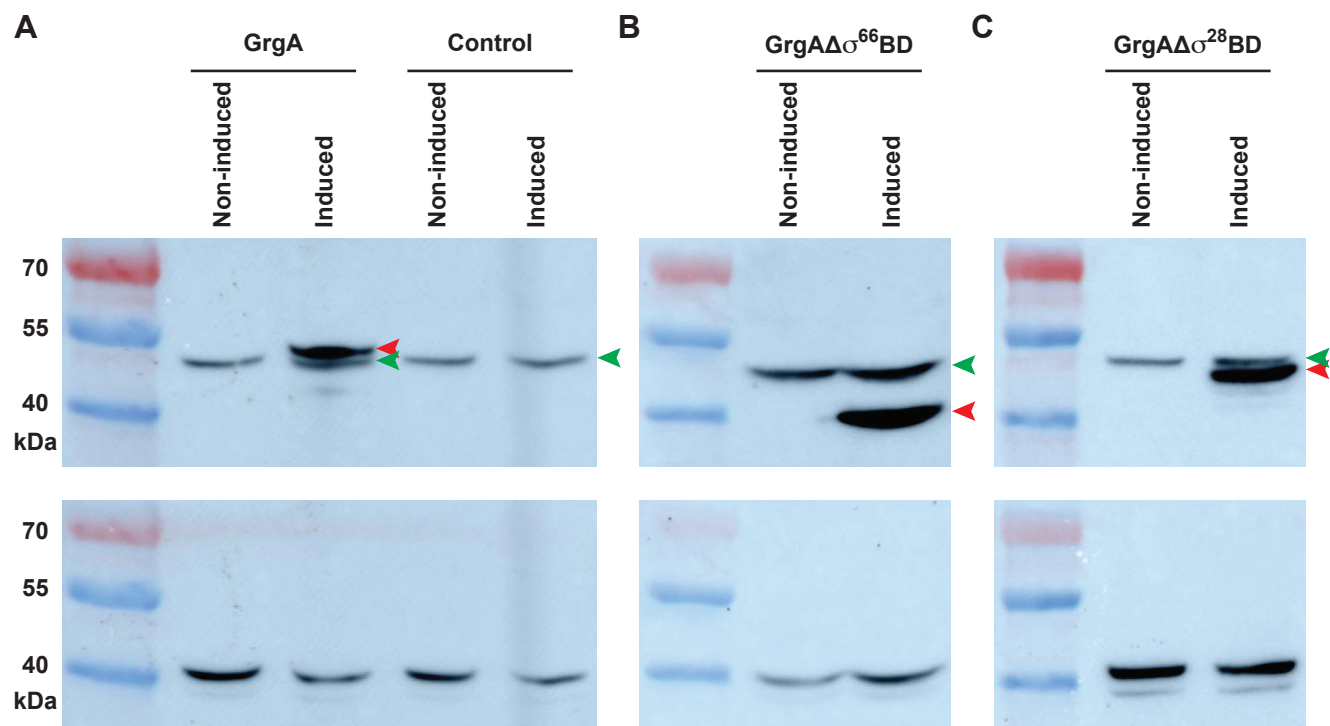

sFig. 1

Supplement: FIG S1 [file msystems.00738-21-sf001.pdf]

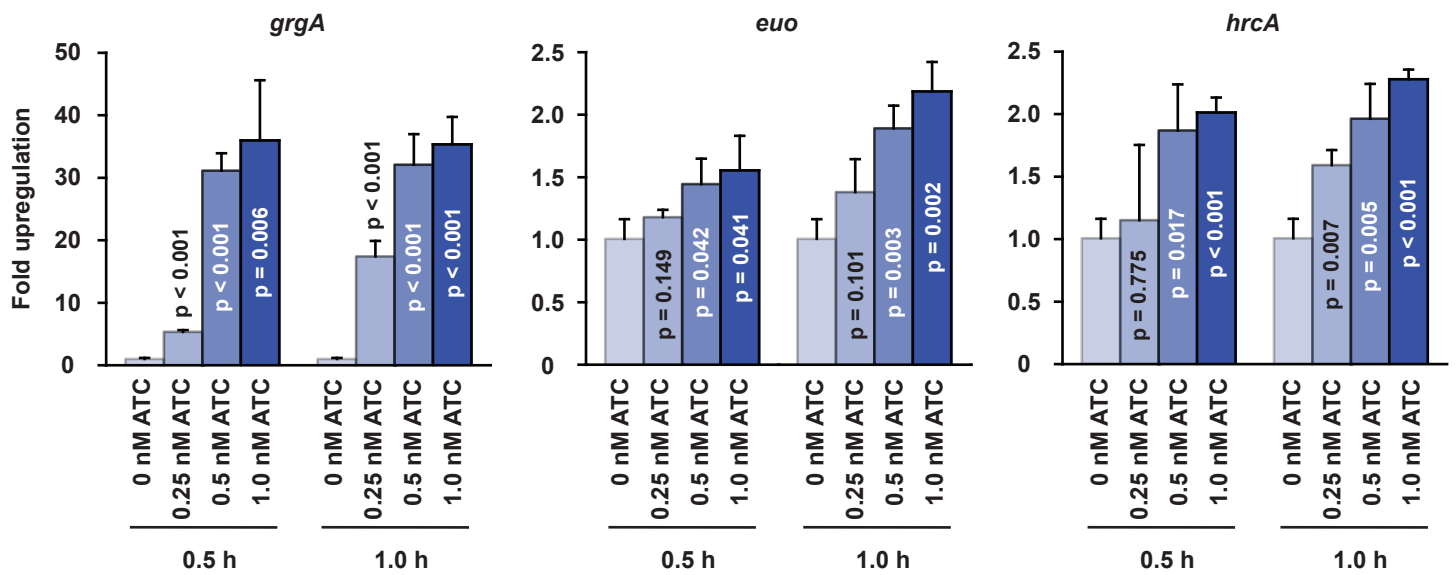

sFig. 2

Supplement: FIG S2 [file msystems.00738-21-sf002.pdf]

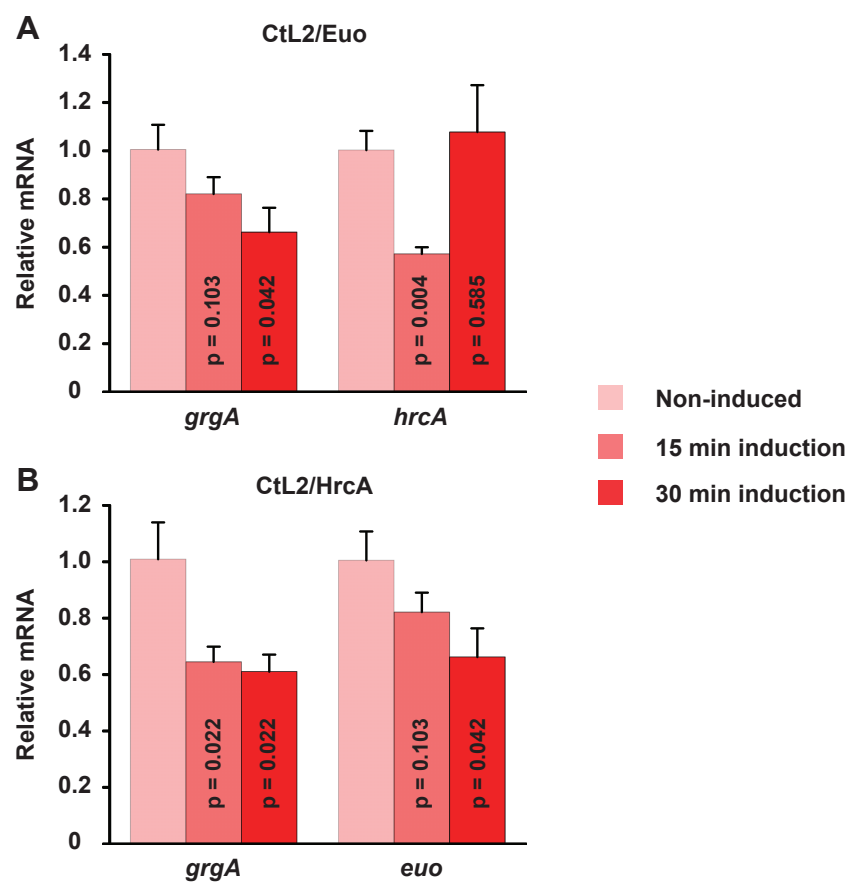

sFig. 3

Supplement: FIG S3 [file msystems.00738-21-sf003.pdf]

1 h

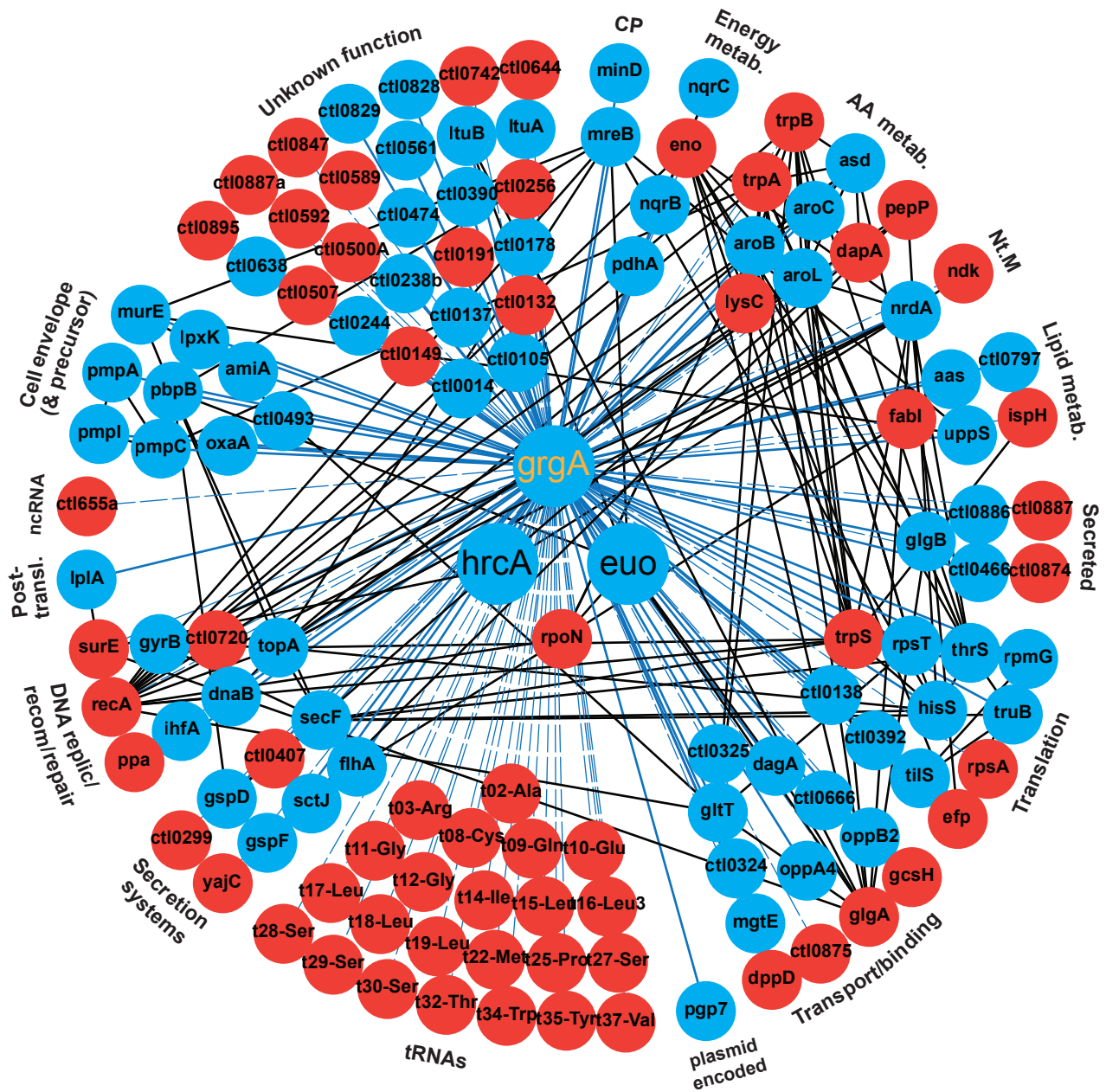

**sFig. 4**

Supplement: FIG S4 [file msystems.00738-21-sf004.pdf]

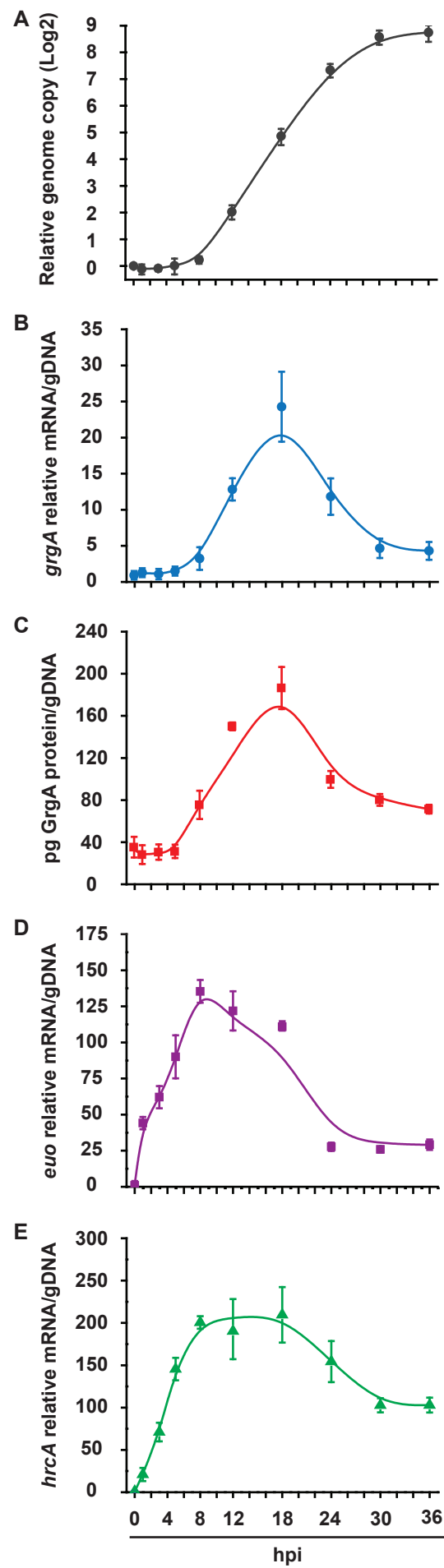

sFig. 5

Supplement: FIG S5 [file msystems.00738-21-sf005.pdf]
